# Use of nutritional supplements and other complementary medicine methods among patients with Parkinson’s disease in Lithuania

**DOI:** 10.3389/fneur.2025.1581590

**Published:** 2025-10-07

**Authors:** Jevgenija Guk, Rūta Kaladytė Lokominienė, Dalius Jatužis

**Affiliations:** Clinic of Neurology and Neurosurgery, Institute of Clinical Medicine, Faculty of Medicine, Vilnius University, Vilnius, Lithuania

**Keywords:** Parkinson’s disease, nutritional supplement, complementary and alternative medicine, CAM, prevalence

## Abstract

**Background and purpose:**

More than half of Parkinson’s (PD) disease patients use nutritional supplements and other methods of complementary medicine (CAM). Only a minority discuss their use with their physician. We aimed to evaluate the prevalence and factors associated with using CAM and nutritional supplements among PD patients in Lithuania.

**Methods:**

The cross-sectional study was conducted at Vilnius University Hospital Santaros Klinikos in 2022. Patients diagnosed with PD were asked to complete the questionnaire about nutritional supplements and CAMs used during the past 12 months.

**Results:**

The data analysis included 169 out of 206 questionnaires. There were 81 (47.9%) males and 88 (52.1%) females. The median age was 66 years old (IQR 59.5–72), and the median duration of PD was 4 years (IQR 2–8). One hundred six patients (62.7%) took nutritional supplements during the last 12 months, and 52 (30.8%) have used some other kind of CAM. Females used such treatment more often than males, respectively, 70.5% vs. 54.3%, *p* = 0.03 for nutritional supplements, and 38.6% vs. 22.2% (*p* = 0.021) for other CAM methods. There were no differences in other sociodemographic and disease-related characteristics between nutritional supplements and other types of CAM users and nonusers. Vitamin D, fish oil, and magnesium were the most frequently used supplements (respectively by 60.4, 51.4, and 54.7% of supplement users). Only 50 (47.1%) nutritional supplements and 35 (67.3%) CAM users discussed using such treatment with their neurologist or treating physician.

**Conclusion:**

Nutritional supplements and CAM therapies are frequently used by PD patients in Lithuania, with a higher prevalence among females. While 67.3% of CAM users discussed their usage with a treating physician or neurologist, only 47.1% of nutritional supplement users did the same. Nevertheless, the latter has a greater potential for interaction with conventional treatments. There is a need to expand the education of general practitioners, neurologists, and PD patients about the indications, effects, and possible side effects of nutritional supplements and other CAM methods, thus raising awareness and encouraging better communication regarding these measures.

## 1 Introduction

Complementary and alternative medicine (CAM) has numerous and varied definitions. It refers to a group of diverse medical and healthcare systems, practices, and products that are not, at present, considered an integral part of conventional medicine (1). It includes such methods as acupuncture, massage, herbal medicine, nutritional supplements, homeopathy, aromatherapy, meditation, yoga, and others (2). This article discusses nutritional supplements independently of other CAM methods, as they were assessed separately. As defined by the European Food Safety Authority (EFSA), nutritional supplements are concentrated sources of nutrients (i.e., minerals and vitamins) or other substances with nutritional or physiological effects that are marketed in “dose” form (e.g., pills, tablets, capsules, liquids in measured doses). A wide range of nutrients and other ingredients might be present in food supplements, including, but not limited to, vitamins, minerals, amino acids, essential fatty acids, fibers, and various plants and herbal extracts (3).

The use of nutritional supplements and other CAM methods has become increasingly popular in Western countries (1, 4). It is more common among females, those with higher education, older adults, and people with chronic diseases (4, 5). More than half of patients with PD take nutritional supplements and other forms of CAM therapy, and about 17% use them specifically to treat their PD symptoms, however, only a minority (11–47%) discuss their use with their treating physician (1, 6, 7). Nutritional supplements may influence the motor and non-motor symptoms of PD by interacting with prescribed medications, which can reduce their effectiveness (5, 8, 9). Certain herbal treatments may also have the potential to induce symptoms resembling parkinsonism (10). However, these interactions are often unknown to patients, and since they are not inclined to discuss nutritional supplements and other CAM methods used with their physician, they cannot obtain this information during the consultation (6, 11, 12). Therefore, it is important to evaluate the use of complementary medicine techniques and nutritional supplements in PD patients and discuss their possible benefits and side effects.

According to the survey conducted from 2021 to 2023, 71.6–78.1% of Lithuanian working-age (18–64 years old) residents used nutritional supplements (13). The number of people in Lithuania who take nutritional supplements has increased by fourfold in the past two decades (14). The use of nutritional supplements and complementary medicine among PD patients in Lithuania has not been studied yet.

The objective of our study was to evaluate the use of nutritional supplements and CAM methods among PD patients with respect to social, demographic, and disease-related factors.

## 2 Methods

### 2.1 Participants

This cross-sectional study was conducted in the Parkinson’s disease center of Vilnius University Hospital Santaros klinikos in 2022. We consecutively included patients diagnosed with idiopathic PD, who were older than 18 years, able to speak and understand the Lithuanian language, and willing to participate in the study. Those participants with severe motor symptoms that may impair their ability to complete the questionnaire did it with the assistance of their caregiver. We excluded patients with atypical parkinsonism and other parkinsonian disorders and those who did not fully complete the questionnaires.

### 2.2 Data collection and tools

The semi-structured questionnaire was distributed to every patient who met the inclusion criteria. The questionnaire was comprised of questions related to sociodemographic issues (e.g., age, sex, residence, educational level, and employment) as well as factors relevant to the health and disease (e.g., perceived physical activity, smoking status, age of motor symptoms onset, and disease duration, Hoehn-Yahr stage). Respondents were asked about the nutritional supplements used in the past 12 months. If respondents reported taking them, they were asked if they were taking any of the 13 individual nutritional supplements (multivitamins, vitamins D, B6, B12, C, E, folic acid, fish oil, potassium, calcium, magnesium, iron, Coenzyme Q10). The list was determined after the available literature review on the use of nutritional supplements in PD patients. We then refined the list based on the insights and experience of the team members regarding the most commonly used nutritional supplements among our patients. Moreover, a free-text box question was included for respondents to report any additional nutritional supplements taken. Supplement users were asked if they take them because of PD, their opinion about the effectiveness of used supplements, and whether they had spoken to a healthcare professional about their supplement use. The questionnaire part on other CAM therapies included questions about their opinion on CAM treatments and whether they use any such measure. Those who reported using CAM in the last 12 months were asked to tick which kinds of the 10 listed techniques they used. Moreover, a free-text box question was included so that respondents could report any additional measures. For each technique used, respondents were prompted to tick whether they were satisfied with it or not. Also, respondents were asked about the reason they used CAM therapies and if they discussed the use with healthcare professionals. The questions included in the questionnaire are provided in the Supplementary material.

Before distributing the questionnaire, the study team members reviewed it for its simplicity, readability, and content. The first 10 respondents who answered the questionnaire were asked about the statements and questions they found difficult to understand. They also provided suggestions for revisions that would help clarify these questions. We then adjusted the questionnaire based on their feedback.

The Institutional Review Board at Vilnius University Santaros clinics approved the protocol.

### 2.3 Statistics

All statistical analyses were conducted using SPSS version 26.0 (IBM Corp., Armonk, NY, United States). Continuous variables are expressed as mean ± SD or otherwise stated. Continuous variables were checked for normal distribution by the Shapiro–Wilk statistic and compared by Student’s t-test when normally distributed or by the Mann–Whitney test for non-normally distributed variables. Categorical variables were compared by χ2-test or Fisher’s exact test as appropriate. The Pearson or Spearman rank correlation coefficients were calculated to test the association between two variables with a normal or non-normal distribution, respectively. A *p*-value <0.05 was considered statistically significant.

## 3 Results

### 3.1 Use of nutritional supplements

**Figure 1:**
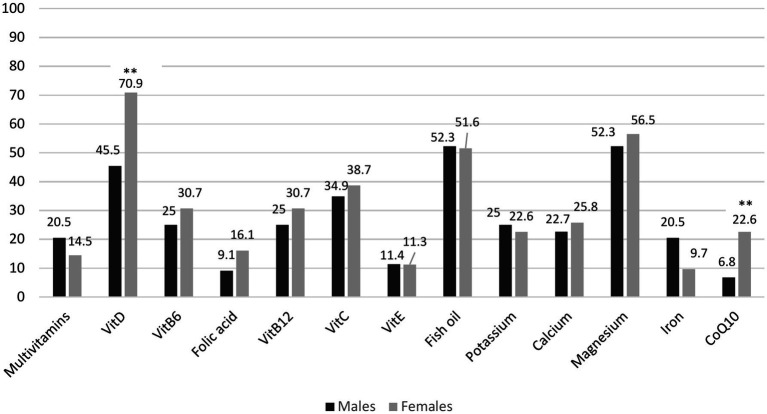
Bar chart illustrating the frequencies (%) of use for different types of nutritional supplements used by males and females. *p < 0.05; **p < 0.01, ***p < 0.001.

One hundred sixty-nine out of 206 respondents met the inclusion criteria and were included in the data analysis. An incomplete questionnaire was the primary reason for exclusion. There were 81 (47.9%) males, age 66 years old (IQR 59.5–72), 119 (70.4%) lived in the city, 53 (31.4%) were employed, and 102 (60.4%) had a college or university education. Parkinson’s disease duration since diagnosis was from 1 to 17 years (median 4 years; IQR 2–8), and more than half of the respondents, 105 (62.1%), had II Hoehn-Yahr stage. The median number of antiparkinsonian medications used was 2 (IQR 1–3). One hundred six patients (62.7%) took nutritional supplements during the previous 12 months. The sociodemographic and disease-related characteristics are shown in Table 1. There were no differences in age, disease duration, age at PD diagnosis, residency, employment, educational level, smoking, physical activity, antiparkinsonian medication use, or Hoehn and Yahr stage between nutritional supplements users and non-users. Females took nutritional supplements more often than males (respectively 70.5 and 54.3%, *p* = 0.030). A total of 28 different nutritional supplements were reported as being used. Figure 1 shows the frequency of different nutritional supplements used by respondents. Only 28 (26.4%) users reported using them specifically for PD. Vitamin D, fish oil, and magnesium were the most frequently used supplements (respectively by 60.4, 51.4, and 54.7% of supplement users). Females used such supplements as Vitamin D and Coenzyme Q10 more often than males (respectively 71.0% vs. 45.5%, *p* = 0.001 and 22.6% vs. 6.8%, *p* = 0.008). Of those patients taking nutritional supplements, only 50 (47.1%) consulted with their general practitioner or neurologist regarding their supplement use (27.2% males and 31.8% females). Forty-one (38.7%) of nutritional supplement users decided to use their supplements by themselves, 23 (21.7%) were advised by family members and friends, and the rest had got advice from a general practitioner or neurologist. Ninety-three (87.7%) of nutritional supplement users reported benefits from supplements, and 64 (60.4%) planned to continue using them.

**Table 1:** Demographic and disease-related characteristics of nutritional supplements users and non-users.

Variable	Users (*n* = 106)	Non-users (*n* = 63)	*p* value
Age, years	68 (59–71)	68 (62–74)	0.075
Sex, *n* (%)			**0.030**
Male	44 (41.5%)	37 (58.7%)	
Female	62 (58.5%)	26 (41.3%)	
Age at PD onset, years	60 (22–65)	64.00 (56–70)	0.150
Disease duration, years	4 (2–8.25)	4 (2–7)	0.688
Residency, *n* (%)			0.241
City	78 (73.6%)	41 (65.1%)	
Rural	28 (26.4%)	22 (34.9%)	
Education, *n* (%)			0.102
University, College	69 (65.1%)	33 (52.2%)	
Lower	37 (34.9%)	30 (47.8%)	
Employment, *n* (%)			0.795
Employed	34 (32.1%)	19 (30.2%)	
Unemployed	72 (67.9%)	44 (69.8%)	
Hoehn-Yahr stage, *n* (%)			0.144
I	18 (16.9%)	8 (12.7%)	
II	68 (64.2%)	37 (58.7%)	
III	18 (16.9%)	16 (25.4%)	
IV	2 (1.9%)	1 (1.6%)	
V	0	1 (1.6%)	
Smoking status, *n* (%)			0.431
Nonsmokers	98 (92.5%)	56 (88.0%)	
Physical activity, *n* (%)			0.126
Physically active	39 (36.8%)	16 (25.4%)	
No physically active	67 (63.2%)	47 (74.6%)	
Antiparkinsonian medications number	2 (1–3)	2 (1–3)	0.273
Dopamine Agonist	63 (59.4%)	32 (50.8%)	0.274
Levodpo (with carbidopa or benserazide)	79 (74.5%)	46 (73.0%)	0.828
MAO-B inhibitors	68 (64.2%)	35 (55.6%)	0.268
Amantadine	26 (24.5%)	17 (27.0%)	0.723
COMT inhibitors	11 (10.4%)	4 (6.3%)	0.373

Values are given as median (interquartile range) or number (percentage). PD, Parkinson’s disease; MAO-B, Monoamine oxidase B; COMT, catechol-O-methyltransferase. Values shown in bold indicate statistical significance at the *p* < 0.05 level.

### 3.2 Use of other CAM methods

**Figure 2:**
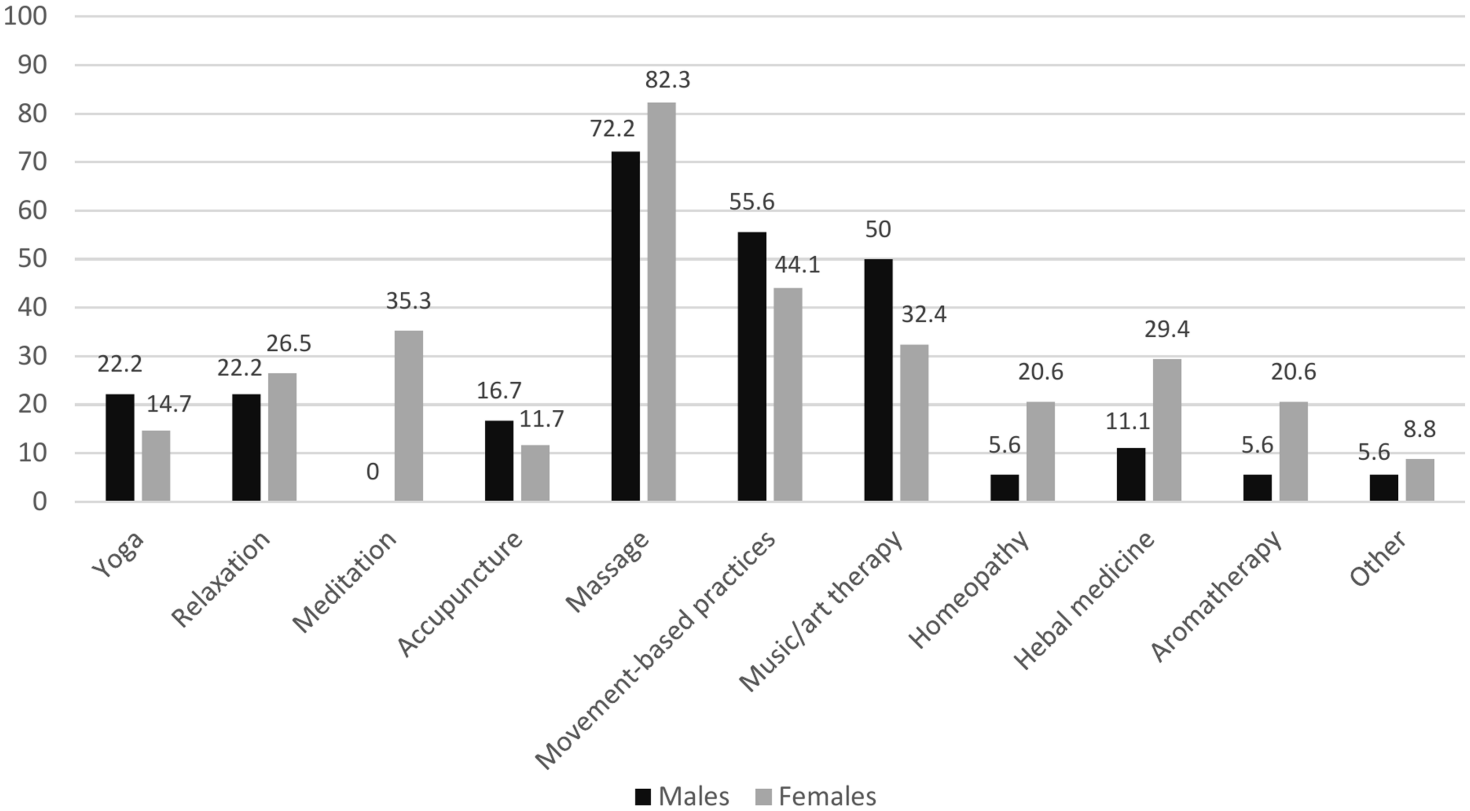
Bar chart illustrating frequencies (%) for different types of complementary therapies used by males and females.

**Figure 3:**
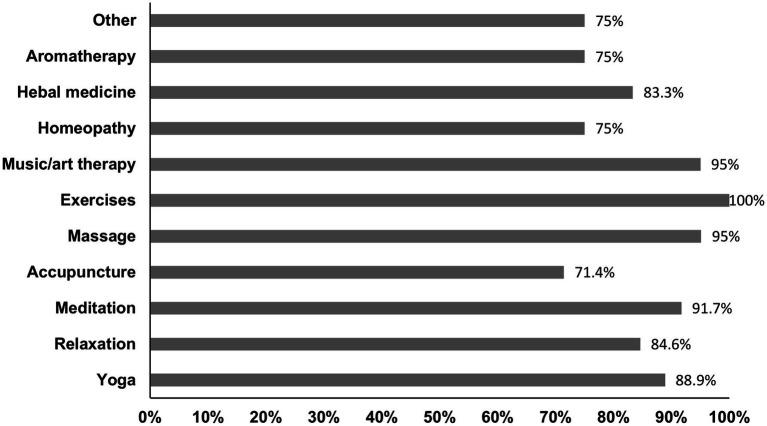
Bar chart illustrating the rate of perceived satisfaction with different CAM methods.

**Figure 4:**
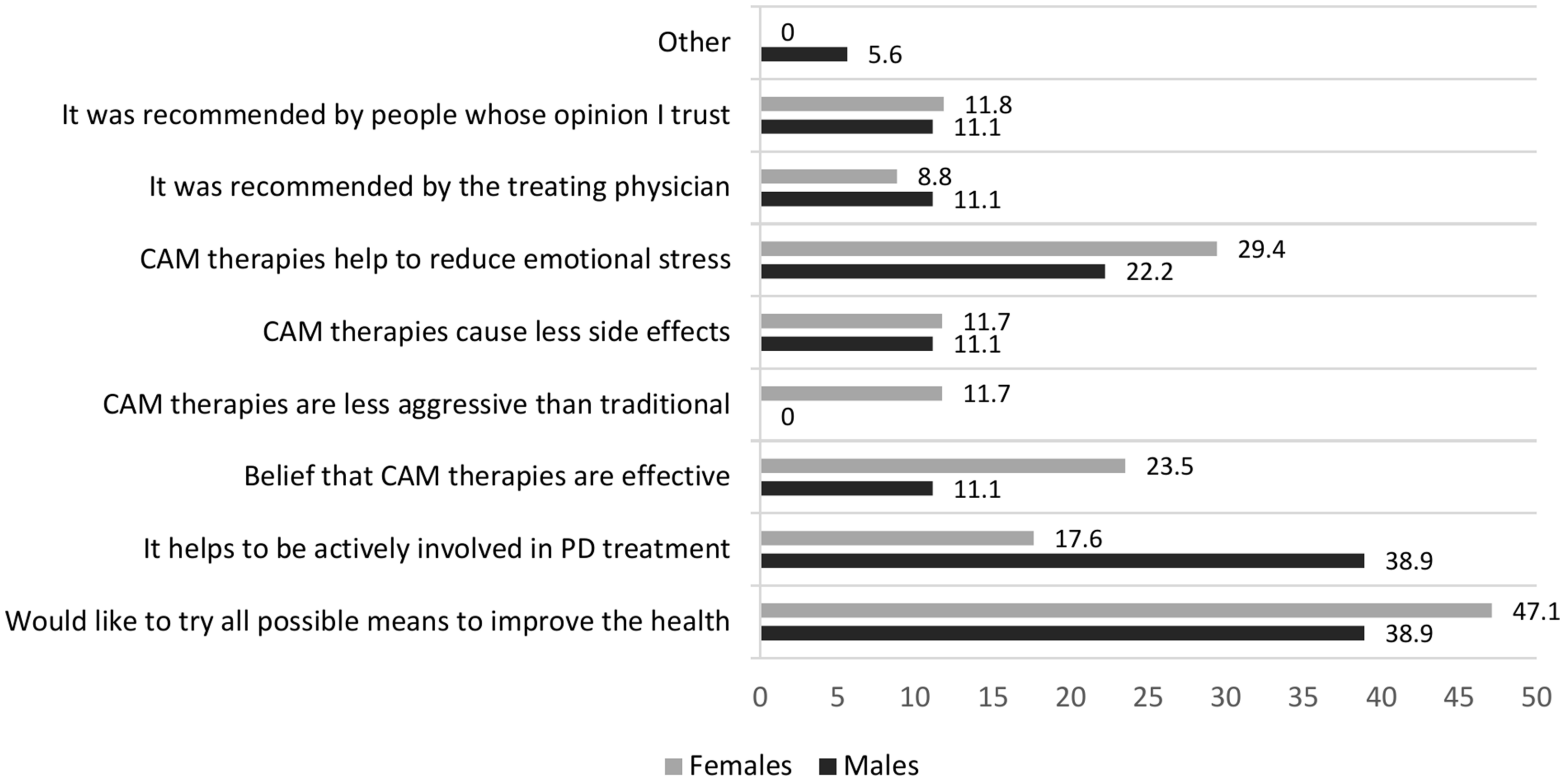
Bar chart illustrating respondents’ most frequently mentioned reason for using CAM methods.

Sixty-four (39.7%) respondents reported a positive attitude toward CAM methods, respectively, 27 (33.3%) males and 37 (42.0%) females, *p* = 0.243. Fifty-two (30.8%) have used some kind of CAM during the last 12 months. Females used this treatment more often than males, respectively 38.6 and 22.2% (*p* = 0.021). There were no differences in age, disease duration, age at PD onset, residency, employment, educational level, smoking status, or Hoehn and Yahr stage between users and non-users. However, those who were more physically active used CAM treatments more often compared to physically inactive respondents (respectively 55.8 and 19.7%, *p* = 0.000). Massage, movement-based practices, and music/art therapy were the most frequently used methods, respectively used by 78.9, 48.1, and 38.5% of respondents. Detailed information about the frequency and types of complementary therapies used by respondents can be found in Figure 2. More than two-thirds of different CAM techniques users were satisfied with the methods they used, with only small differences between therapies. Figure 3 provides the detailed perceived satisfaction rate for each CAM method. Thirty-five (67.3%) individuals (61.1% males and 70.6% females) discussed CAM use with a treating physician or neurologist. Among the most common reasons to use CAM were the wish to try all possible means to improve health (44.2%) and the belief that such methods help to reduce emotional stress (26.9%). Figure 4 provides detailed information on respondents’ motivations for using complementary medicine.

## 4 Discussion

The study found that 62.7% of surveyed PD patients used nutritional supplements, and 30.8% used different types of other CAMs. This is less than in the general population in Lithuania. According to the survey conducted from 2021 to 2023, 71.6–78.1% of Lithuanian working-age (18–64 years old) residents used nutritional supplements (13). This discrepancy might be explained by the possibility that PD patients are aware of the commonly occurring negative attitude to CAM expressed by many physicians who prescribe traditional PD treatment, so patients may thus withhold this information or resort to a variety of strategies to conceal their CAM use (15). To prevent misunderstandings and identify the actual issues, physicians should proactively inquire about a patient’s use of nutritional supplements and other CAM methods. It is important to approach this discussion without negative connotations and to provide evidence-based information regarding the necessity, effectiveness, and potential side effects of these measures.

According to studies conducted in the United States, more than two-thirds of PD patients use different nutritional supplements and it is more often than in the general population (6, 7, 16, 17). However, studies conducted in Europe indicate a lower use rate. The study conducted in Sweden showed that only 20% of PD patients used CAM nutraceuticals. While nutritional supplements are not addressed separately in the paper, they could fall under “other drugs from the health drug store” (15). According to data obtained from the Netherlands, 36% of PD patients used natural health products to alleviate PD-related symptoms, but even 71% of PD patients were interested in learning more about such products (11). Our results show that females with PD took nutritional supplements more often than males. Similar results were obtained by Lökk et al. (15). However, no differences in sex between nutritional supplement users and nonusers were found in other studies (6, 11, 16, 17). Our findings may be related to the fact that in Lithuania, the use of nutritional supplements is generally more common among women than men (14). The studies conducted in the United States, Europe, and China generally found that females used different CAM more frequently than males (4, 18–20). Such discrepancy may be due to differences in values and personality traits, such as risk-seeking behavior, between males and females (4).

There was no association between nutritional supplement use and age, disease duration, age at PD onset, residency, employment, educational level, smoking, physical activity, or Hoehn and Yahr stage between nutritional supplement users and non-users. Similar results were obtained by Wolfrath et al., and Ferguson et al. (6, 16). However, Rajendran et al. found that complementary therapy users were younger, had a younger age of PD onset, higher income, and educational level (17). In the study by Diadhiou et al., only younger age was significantly associated with the use of herbal remedies (11). According to a study conducted in Europe, the use of CAM in general is more prevalent among people in middle age, those with health issues, those in higher sociodemographic groups, and those with greater levels of education (4). People who live in cities, have greater levels of education, and are younger consume nutritional supplements more frequently, according to the Nutritional Habits of the Lithuanian Population Survey (14). Our study’s findings may indicate that, independent of sociodemographic and disease-related characteristics, having Parkinson’s disease (PD) may predict the usage of nutritional supplements. Patients with other chronic neurological conditions such as multiple sclerosis, dementia, and amyotrophic lateral sclerosis frequently take nutritional supplements (respectively 77, 46, and 43.9%) (21–23). The reason could be the presence of the struggling and debilitating symptoms of PD and the limitations of current conventional treatment. Previous studies found that chronic diseases, unmet medical needs, and dissatisfaction with health systems are related to more frequent CAM use (1, 4). Therefore, the physician should determine which symptoms the patient is using nutritional supplements to relieve and discuss the possibility of treating these symptoms with conventional methods, and the potential effectiveness of nutritional supplements and other CAM approaches to alleviate both motor and non-motor symptoms of PD and their impact on the disease progression.

Vitamin D, fish oil, and magnesium were the most frequently used supplements in our study. Our study did not determine which symptoms or conditions prompted the subjects to use specific nutritional supplements. Furthermore, information on comorbidities was not gathered, making it impossible to understand the respondents’ reasons for using particular nutritional supplements. Previous studies found vitamin E to be the most frequently used supplement by PD patients (17). Ferguson et al. found that vitamin D, multivitamins, and Vitamin B12 were most frequently used by PD patients (16). The most recent study in the United States found that the most used supplements among PD patients were the vitamins D, C, and B12, while the most often taken herbal remedies in the Netherlands were coffee, cannabis, and turmeric (7, 11). Our data shows that only 25% of males and 30% of females use vitamin B12 supplements. The lack of routine testing for vitamin B12 levels in Lithuania may explain why elderly individuals and patients with PD often remain unaware of their deficiency. As a result, they do not receive the necessary supplements.

In our study, only 26.4% of patients reported using supplements for PD; the rest of the users had used them for other reasons. Our findings are consistent with those of the Wolfrath et al. trial, where 26% of participants used supplements to manage PD symptoms (6). Rajendran et al. found that even 40% of respondents specifically used nutritional supplements and other kinds of complementary therapies for PD treatment, but in this study, supplements and other CAM measures were evaluated together (17).

The potential clinical and neuroprotective benefits of several popular nutritional supplements included in this survey have been investigated, but the body of research remains limited in supporting the use of any of these nutritional supplements to improve PD symptoms and progression.

Vitamin D plays a crucial role in maintaining calcium and phosphorus balance as well as bone metabolism (24). Experimental *in vitro* and animal models showed that it lowers the synthesis of pro-inflammatory cytokines, boosts antioxidant concentrations, protects against excitotoxicity, and possesses neuroprotective qualities. A high frequency of vitamin D deficiency was detected in PD patients compared with the control population, and some authors observed the relationship between low serum vitamin D levels and severity of PD motor symptoms assessed by UPDRS scores and non-motor symptoms like depression and cognitive impairment, and disease progression. Still, there is very little data to support vitamin D supplementation for reducing clinical manifestations and disability in patients with PD (24, 25). According to the International Parkinson and Movement Disorder Society evidence-based medicine review, there is not enough evidence to support the use of vitamin D to prevent or slow the progression of the disease, but it is still being investigational for this purpose (26). However, the patients with PD usually suffer from osteoporosis, leading to an elevated risk of hip fractures owing to lower bone density along with low calcium (27). Moreover, the prevalence of PD increases with age, particularly affecting those above the age of 60 years (28). Meanwhile, the risk of vitamin D deficiency increases among older people and postmenopausal women (29, 30). Also, the risk is higher among northern latitude residents (31). Timely detection, treatment, and prevention of vitamin D deficiency are crucial for older individuals, particularly those at higher risk. According to the updated recommendations from the Lithuanian College of Family Physicians, vitamin D should be administered to treat vitamin D deficiency and as a preventive measure for individuals in at-risk groups, such as those who are obese or older than 75 years. Lithuanian residents are advised to take additional vitamin D supplements during the months with insufficient sunlight, from September to May. In the other months, supplementation is also recommended, especially for high-risk groups susceptible to vitamin D deficiency (32).

Fish oil contains polyunsaturated fatty acids (PUFAs). Omega-3 PUFAs possess neuroprotective, antioxidant, and anti-inflammatory qualities in animal models, but there is no strong clinical evidence that PUFAs reduce PD progression or have any symptomatic effects (33). According to the International Parkinson and Movement Disorder Society evidence-based medicine review of non-motor symptoms treatment, omega-3 fatty acids have insufficient evidence of treating depressive symptoms in PD patients, but have an acceptable risk without specialized monitoring and are considered investigational (34).

Coenzyme Q10 (CoQ10) is an essential component in the mitochondria’s electron transport chain that contributes to oxidative phosphorylation and cellular energy generation. Decreased levels of CoQ10 were found in serum and other tissues of PD patients. CoQ10 shows antioxidative properties and prevents the loss of dopaminergic neurons in animal PD models. Despite the possible beneficial effects of CoQ10 administration, the lack of important adverse effects, and the improvement in PD symptoms suggested by several studies, data from meta-analyses of randomized clinical trials did not suggest the general usefulness of this therapy in PD patients (35). A dose-dependent neuroprotective effect was demonstrated in earlier randomized, placebo-controlled trials; however, meta-analyses revealed no disease-modifying benefits. According to the MDS review of an evidence-based update on treatments for the motor symptoms of PD, CoQ10 is classified as non-efficacious and not useful to prevent or delay disease progression (26). Following the British National Institute for Health and Care Excellence (NICE) guidelines, CoQ10 should not be prescribed as a neuroprotective therapy for patients with PD (36).

A 2017 cross-sectional analysis of modifiable variables in the Parkinsonism (MVP) study using patient-reported outcomes in Parkinson’s Disease (PRO-PD) scale showed that coenzyme Q10 and fish oil were associated with better outcomes (37). However, this study aimed to evaluate the association of nutraceutical use with a subjective outcome measure. Subjective measures are susceptible to biases; they are less reliable and accurate than objective measures.

Magnesium is one of the essential microelements that play a crucial role in energy and substrate metabolism, cell signaling, and homeostasis processes (38). It is typically recommended for the treatment of arrhythmias and as a supplement to enhance glycemic control and facilitate muscle cramps, hypertension, constipation, sleep quality, mood, and anxiety (39–42). Studies conducted on animals and *in vitro* revealed that magnesium deficiency was linked to oxidative stress, neuroinflammation, *α*-synuclein aggregation, and dopaminergic cell death (38). However, there is currently no evidence that magnesium intake can lower PD risk or improve motor or non-motor symptoms (38). PD patients can use magnesium to relieve muscle cramps, constipation, to improve sleep and mood. But it is important to consider that concomitant use of levodopa and magnesium oxide can decrease the levodopa plasma concentration, resulting in worsening motor symptoms (9).

Vitamins are well known for their anti-inflammatory and antioxidant properties. It has been suggested that B vitamins, such as folate, vitamin B6, and vitamin B12, can protect against PD development (43). In preclinical studies, they showed antioxidant properties, reduced neurotoxic homocysteine levels, promoted dopamine production, and modulated leucine-rich repeat kinase 2 (LRRK2). However, according to the large prospective observational study results published in 2023, folate or vitamin B6 did not reduce PD risk in the evaluated population, and vitamin B12 had moderate support for a possible protective effect on the development of PD (44). However, Vitamin B12 deficiency is common among older adults due to inadequate dietary intake and impaired absorption, resulting from atrophic gastritis, bacterial overgrowth, or prolonged use of medications for other health conditions, and is even more prevalent in PD patients (45, 46). Also, vitamin B12 deficiency is linked to neurological issues such as dementia, ataxia, paresthesia, paralysis, gait abnormalities, peripheral neuropathy, psychosis, and mood disorders (46). Vitamin B12 supplementation may be necessary for individuals with chronic conditions such as atrophic gastritis, neuropathies, and others. It is essential to assess vitamin B12 deficiency in at-risk individuals and provide appropriate supplementation.

Our results show that only 47.1% of patients using supplements consulted with their physician about using them. Similar results have been found in other studies: 39 to 47% of PD patients who used nutritional supplements or alternative therapies consulted their treating physician about using these measures (6, 11, 17). However, Ferguson et al. revealed that even 70.8% of respondents consulted with healthcare professionals about supplement usage. One possible explanation for this rise is that healthcare professionals are now more aware of the use of nutritional supplements by PD patients (since this was the third study of this kind to be done in the United States), which has encouraged them to talk to their patients about it. The other explanation could be that PD patients became more aware that nutritional supplements may interfere with other medications (16). Only 4% of respondents to a 2006 survey by Wolfrtah et al. were aware of potential medicine and nutritional supplement interactions, but 39% of consumers of herbal remedies were aware of such interactions, according to a 2024 study by Diadhiou et al. (6, 11). It is crucial to discuss with the patient the use of nutritional supplements and possible interactions with antiparkinsonian medication because some of them can diminish the effectiveness of such treatment. For example, concomitant use of iron and magnesium oxide with levodopa can decrease its absorption (8, 9). Herbal remedies are often considered as safe and natural; however, they can cause side effects and interact with antiparkinsonian medications. Components of ginseng (*Withania somnifera* L.), Ginkgo biloba L., and St John’s Wort (*Hypericum perforatum* L.) have been demonstrated to influence the reuptake of neurotransmitters, such as norepinephrine, dopamine, and serotonin, and may predispose to serotonin syndrome (47).

Although 39.7% of our respondents said they had a positive attitude toward complementary medicine, only 30.8% reported using or having previously used CAM methods. Most patients used CAM in conjunction with, rather than as an alternative to traditional treatment. Females used such treatment more often than males. There were no other sociodemographic differences between users and non-users in our study. However, those who were more physically active used complementary medicine more frequently compared to physically inactive respondents. According to previous studies, CAM use for PD treatment ranges from 25.7 to 85% in different countries and is more prevalent in Asia than in Europe and the Americas due to historical and regional differences (1, 12, 48). Donley et al. also found that females were more likely than males to use CAM (12). Other research identified correlations between the use of CAM and female sex, age, education level, higher incomes, perceived poor health, and rural areas. However, not all the investigations found these correlations (1). Physical activity has been identified as an independent factor influencing the use of CAM in the general population (49). It is possible that individuals who are physically fit tend to be more health-conscious and, therefore, may prefer alternative healthcare options (1). This preference may be related to a strong health locus of control, as individuals with a health locus of control are more likely to engage in behaviors that improve or maintain their daily functioning and overall health. One such behavior could be the use of CAM (15). Massage, movement based practices, and music/art therapy were the most frequently used methods of the other CAM therapies among our respondents. Massage was the most frequently used CAM treatment among PD patients in the United States and the United Kingdom (1, 12, 48). Acupuncture, aromatherapy, yoga, Thai chi, oriental medicine, and relaxation techniques were among the most popular therapies listed by PD patients in other countries (1). However, the spectrum of CAM use may be greatly influenced by the cultural traditions and coverage by the health-care system of different countries. More than two-thirds of users of different techniques were satisfied with the methods they used. Donley et al. reported that the greater part of the treatments listed in their survey had extremely high perceived efficacy (>50% of patients thought they were effective). The perceived effectiveness of different CAM methods was about 40% in other studies, but most CAM users in Sweden reported no improvement or only some improvement (1, 15). The placebo effect may account for the perceived high effectiveness of CAM measures (50). Numerous clinical trials have demonstrated he responsiveness of PD patients to placebo interventions, which can elicit meaningful changes in both subjective experiences and measurable health outcomes (51, 52). According to our survey, the wish to try every possible means to improve health and the belief that CAM treatment helps to reduce emotional stress were among the most popular reasons for using CAM. Similar reasons were indicated in Lökk et al. study conducted in Sweden (15).

Donley et al. found that people use different CAMs to reduce such symptoms as stress, tremor, muscle tightness, stiffness, and anxiety, and improve balance and mood (11). The willingness to improve PD motor symptoms, augment the effects of prescribed medications, and management of various non-motor symptoms such as fatigue, pain and constipation were among the most common reasons in other studies (1). Other reasons put out by other researchers include the patient’s philosophical views, their belief in the treatment, and the perceived ineffectiveness of conventional treatment for these symptoms (1). Our findings showed that those who used other CAM techniques were more likely to talk to healthcare professionals about their use than people who took nutritional supplements (67.3% versus 47.1%, respectively). One possible explanation is that the part of CAM techniques (e.g., massage) included in our survey is usually prescribed by family physicians or physiotherapists.

We found that nutritional supplement use is more prevalent than other CAM methods among PD patients in Lithuania (63% and 30.8% respectively). The possible reason for such a difference could be the cost and limited coverage by the health care system. The cost of CAM treatments is higher than that of commonly used nutritional supplements. Such CAM techniques, like massage and physiotherapy, receive limited coverage from the Lithuanian health care system, and others mentioned in our survey are not covered at all.

CAM has only a few published reports containing scientific evidence. According to the MDS review of the evidence-based update on treatments for the motor symptoms of PD, physiotherapy (exercises including treadmill, aerobic, and strengthening/balance exercises) is considered to be likely efficacious and clinically useful. Formalized patterned exercises, including dance and Tai Chi, despite insufficient evidence, are considered “possibly useful” in clinical practice (26). Acupuncture has been extensively investigated for treating several different disorders, including PD. Even though studies show a positive effect on improving motor and non-motor symptoms in PD, the great discrepancies regarding the studies’ design and methodology make it difficult to draw generalized conclusions, so acupuncture remains “investigational” for motor symptoms treatment in PD (26, 34, 53).

Study limitations include a cross-sectional design, a small sample size, and possible selection bias due to all patients being recruited from one center. Although there are only 3 tertiary movement disorders centers in Lithuania, patients from our center were considered representative of Lithuanian PD patients as they come from different regions of the country. Additionally, patients who consent to fill out the questionnaire might be more interested in using CAM techniques and nutritional supplements compared to the general PD patient population, which could add some bias. A potential source of bias in our study is the generation of the list of nutritional supplements and other CAM methods. The list was created on review of the available literature, as well as insights and experience from team members. To mitigate this potential bias and ensure that we do not miss any nutritional supplements or other CAM methods, we included a free-text box for participants to provide additional information. Moreover, as in many questionnaire studies, there is always a risk of recall bias. The short questionnaire did not allow for a detailed description of parameters like the frequency and duration of use, the dosage, and the specific PD symptom addressed. Additionally, the data regarding motor and non-motor symptoms, the Unified Parkinson’s Disease Rating Scale, and Quality of Life scale scores were not collected, which limited the ability to correlate these indicators with the use of nutritional supplements and other CAMs. We gathered only general information about whether respondents benefited from nutritional supplements, without indicating which specific supplements provided those benefits and what kind of benefits they felt. The questionnaire also omitted questions on income, although income can influence the patient’s capacity to buy costly CAM techniques and nutritional supplements that are generally not reimbursed by the healthcare system. So more detailed evaluation addressing these limitations should be the topic of further research.

In conclusion, the results of this study revealed the relatively high prevalence of nutritional supplements and other CAM techniques used among PD patients. The number of female users was higher. There was no association between other sociodemographic and disease-related factors between users and non-users. Only 47.1% nutritional supplement users and 67.3% CAM users discuss using such measures with their neurologist or treating physician. This difference could be because CAM practices are often considered as more significant interventions for managing. Therapies such as acupuncture, tai chi, and massage are typically regarded as treatment strategies that involve professional guidance, leading patients to seek validation or advice from their clinicians. In contrast, nutritional supplements are readily available over-the-counter and are often marketed as safe or routine, which may diminished patients’ perceived need to disclose their use of these supplements. Also, the lack of communication may stem from a lack of knowledge among both physicians and PD patients regarding how nutritional supplements and CAM methods work, their side effects, and interactions with medications prescribed for PD. There is a clear need to enhance education for physicians and patients about these treatments, including their benefits and potential risks. These measures can help improve the communication between them regarding the use of nutritional supplements and other CAM methods. Our research findings should encourage neurologists to specifically inquire about the use of such measures in their clinics. Nutritional supplements are widely accessible and do not require a medical prescription, are simple to use, and are frequently thought to be “healthier” than conventional medicine. However, there is no scientifically based evidence regarding their efficacy and benefits. One should remember that conventional medicine should ensure a safe and evidence-based treatment. It is essential to discuss the existing evidence for nutritional supplements and other CAM methods with a patient, caution them against using potentially ineffective or harmful therapies, and discuss possible side effects and potential interactions between such substances and conventional medications.

## Data Availability

The raw data supporting the conclusions of this article will be made available by the authors, without undue reservation.
