# Supplementary material for: Use of nutritional supplements and other complementary medicine methods among patients with Parkinson’s disease in Lithuania
Source: Front Neurol. 2025 Oct 7;16:1581590. doi: 10.3389/fneur.2025.1581590 (PMC12537411; doi:10.3389/fneur.2025.1581590)
Supplement: Supplementary file 1 [file Data_Sheet_1.docx]

Table S1. Questions and categories with relevant response options included in the nutritional supplements and other complementary medicine use in patients with Parkinson’s disease questionnaire.

| 1. Age, years | 2. Sex: Male/Female |
| --- | --- |
| 3. Residence: City/Rural | 4. Highest level of education completed: University or College/Lower (Primary school/Secondary school) |
| 5. Current employment status: Employed/Unemployed (unemployed, retired due to age, retired due to disability) | 6. Smoking status: No (Never smoker/Former smoker)/Current smoker |
| 7. How do you rate your physical activity? Physically active (Regular Exercises >3 times a week/Regular exercises 1-2 times a week)/No physically active (Irregular Exercises less than 1 time a week /Any Exercises) | 8. Age at PD diagnosis, years |
|  | 9. Hoehn-Yahr stage |
| 10. List the medications you take to treat PD. | 11. Do you take nutritional supplements: Yes/No |
| 12. What nutritional supplements have you taken in the last 12 months? Multivitamins/Vitamin D/Vitamin B6/Vitamin B12/Folic Acid/Vitamin C/Vitamin E/Fish oil/Potassium/Calcium/Magnesium/Iron/Coenzyme Q10/Other (Specify) | 13. Who recommended the nutritional supplements you are currently taking? General practitioner or other physician/ Neurologist/Family members or friends/My decision/Other (Specify) |
| 14. Do you take dietary supplements for Parkinson's disease? Yes/No | 18. Have you discussed the use of nutritional supplements with your general practitioner, other physician, or neurologist? Yes/No |
| 19. Do you feel any benefit from the nutritional supplements you consume? No/Yes | 20. Are you planning to keep taking the supplements you are currently using? Yes/No/Haven't decided yet. |
| 21. What is your opinion about other CAM methods? Positive/Negative/Don't know | 22. Have you used any cam method in the last 12 months? Yes/No |
| 22. What CAM methods have you used in the last 12 months?** Yoga/Relaxation/Meditation/Acupuncture /Massage/Movement-based practices like Tai-Chi, Dancing, Stretching exercises, and other similar activities/Music or art therapy/ Homeopathy/Herbal medicine/Aromatherapy/ Other (Specify) | 23. What was the reason for using CAM methods? I would like to try all possible means to improve the health/ It helps to be actively involved in PD treatment/Belief that CAM therapies are effective/ CAM therapies are less aggressive than traditional/ CAM therapies cause fewer side effects/ CAM therapies help to reduce emotional stress/ It was recommended by the treating physician/ It was recommended by people whose opinion I trust/ Other |
| 24. Were you satisfied with the method you used? Yes/No *** | 25. Have you discussed the use of CAM methods with your general practitioner, other physician, or neurologist? Yes/No |

*In case of larger categories, the responses are provided in brackets. **The questionnaire clarified the term "other CAM methods" and provided some examples. ***This question inquired about the use of each CAM method.
